# Design of a randomized, non-inferiority trial to evaluate the reliability of videoconferencing for remote consultation of diabetes

**DOI:** 10.1186/1472-6947-14-11

**Published:** 2014-02-14

**Authors:** Farhad Fatehi, Melinda Martin-Khan, Leonard C Gray, Anthony W Russell

**Affiliations:** 1 Centre for Online Health, The University of Queensland, Brisbane, Australia; 2 School of Advanced Technologies in Medicine, Tehran University of Medical Sciences, Tehran, Iran; 3 Centre for Research in Geriatric Medicine, The University of Queensland, Brisbane, Australia; 4 Princess Alexandra Hospital, Brisbane, Australia; 5 School of Medicine, The University of Queensland, Brisbane, Australia

**Keywords:** Diabetes, Telemedicine, Remote consultation, Videoconferencing, Video teleconsultation, Video consultation, Video visit

## Abstract

**Background:**

An estimated 366 million people are living with diabetes worldwide and it is predicted that its prevalence will increase to 552 million by 2030. Management of this disease and its complications is a challenge for many countries. Optimal glycaemic control is necessary to minimize complications, but less than 70% of diabetic patients achieve target levels of blood glucose, partly due to poor access to qualified health care providers. Telemedicine has the potential to improve access to health care, especially for rural and remote residents. Video teleconsultation, a real-time (or synchronous) mode of telemedicine, is gaining more popularity around the world through recent improvements in digital telecommunications. If video consultation is to be offered as an alternative to face-to-face consultation in diabetes assessment and management, then it is important to demonstrate that this can be achieved without loss of clinical fidelity. This paper describes the protocol of a randomised controlled trail for assessing the reliability of remote video consultation for people with diabetes.

**Methods/Design:**

A total of 160 people with diabetes will be randomised into either a Telemedicine or a Reference group. Participants in the Reference group will receive two sequential face-to-face consultations whereas in the Telemedicine group one consultation will be conducted face-to-face and the other via videoconference. The primary outcome measure will be a change in the patient’s medication. Secondary outcome measures will be findings in physical examination, detecting complications, and patient satisfaction. A difference of less than 20% in the aggregated level of agreement between the two study groups will be used to identify if videoconference is non-inferior to traditional mode of clinical care (face-to-face).

**Discussion:**

Despite rapid growth in application of telemedicine in a variety of medical specialities, little is known about the reliability of videoconferencing for remote consultation of people with diabetes. Results of this proposed study will provide evidence of the reliability of specialist consultation offered by videoconference for people with diabetes.

**Trial registration number:**

Australian New Zealand Clinical Trials Registry ACTRN12612000315819.

## Background

Diabetes Mellitus (DM) is a common disease with increasing prevalence in many countries. More than 366 million people are estimated to have diabetes worldwide and it is projected to increase to 552 million by 2030, affecting 9.9% of the global adult population [1]. Managing diabetes and its complications is very costly, and creates a substantial burden on the health care economy. There is no cure; instead optimal glycaemic control is required to minimize complications [2]. However, less than 70% of people with diabetes are achieving target glycaemic control, demonstrating that effective disease management for people with diabetes remains a challenge [3,4]. For some patients, particularly in rural areas, not achieving target glycaemic controls is at least in part due to poor access to qualified health care providers [5,6]. In response to the growing demand for health care and a decreasing availability of health care providers, information and communications technology (ICT) has shown potential to improve the accessibility of health care services and to reduce costs of health care delivery [7].

Telemedicine is the provision of medical and health services remotely using information and communication technology [8]. The telemedicine interactions are generally divided into two categories: synchronous (occurring in real time such as videoconferencing) and asynchronous (store-and-forward solutions such as transmission of a blood glucose level from a glucometer to a health centre).

Asynchronous (store-and-forward) telemedicine has been successfully implemented in the medical specialities such as pathology [9], radiology [10] and dermatology [11] where real time exchange of information between health care providers and consumers is not essential. In contrast, synchronous telemedicine requires both parties to interact with each other in real time using communication technology. Among the synchronous telemedicine solutions, videoconferencing – real time exchange of voice and image - is becoming popular through rapid achievements in digital communication technology. Video teleconsultation has been used in a wide range of disciplines from emergency medicine [12] to mental health [13], but it has been emphasized that most existing disciple-specific studies cannot be generalized to other tele-medical contexts [14].

In a systematic review on synchronous and asynchronous teleconsultation for diabetes care, Verhoeven et al. suggested that both teleconsultation solutions are feasible, cost-effective and reliable for delivering diabetes care. However they identified a lack of high quality studies and diversity in the included studies [15]. Several studies have reported results of using videoconferencing for diabetes care. However, almost all used videoconferencing for behavioural therapy including diabetes education, self-management training, nutrition counselling, and collaborative goal setting [16]. The accuracy of videoconferencing for specialist telediagnosis and assessment of selected diseases has been studied (e.g., Alzheimer’s disease) [17], but there is no published study on the reliability and accuracy of videoconferencing for clinical consultation with regard to medical specialist evaluation and management of diabetic patients.

If a doctor is able to assess and manage a diabetic patient via videoconference with a similar level of reliability as a face-to-face consultation, the medical profession could have confidence in including video consultation as a regular aspect of their clinical care. A clinician who regularly sees patients for evaluation of their diabetic management may choose to substitute some of the regular face-to-face consultations with a video consultation. This has important implications for patients isolated by either their physical location (e.g., rural communities) or their function (e.g., disabled older people in aged care facilities) and paves the way for at least some specialist consultation for people living in rural or remote areas to avoid the expense and inconvenience of long distance travel. It may also increase the opportunities for some people to receive advice in situations where they previously may have not, due to an inability to travel. This protocol describes a research project that will evaluate the reliability of clinical decisions made during a diabetic patient consultation via videoconference.

Typically the aim of a Randomized Controlled Trial (RCT) is to identify if superiority exists between two or more parallel groups to guide decision making, for example whether to replace a current medication or procedure with a new one. However, an alternative analytical approach is required when the aim is to identify if an innovation is suitable to replace an existing process at the same level of efficacy [18]. Such methodology is referred to as a non-inferiority trial. A priori defined level of clinically acceptable variation between the two modes of delivery is used to determine the outcome. This study is a non-inferiority trial comparing the clinical outcomes of video consultations against those of conventional face-to-face consultations.

### Aims and objectives

In this study we are seeking to identify if telemedicine is a reliable vehicle for providing specialty consultation for people with diabetes using videoconferencing, with the implication that it would be useful to utilise when usual care is either not available or difficult to deliver. The aim of the study is to test the level of clinical agreement achieved using specialist to patient videoconferencing (VC) compared with face-to-face (FTF) consultation. To place this level of agreement in context, it will also establish the level of agreement among specialists using face-to-face consultation. This approach enables the variation in clinical decision making among clinicians to be identified, and thus to be differentiated from the effect of the VC mode of service delivery.

This study will test the hypothesis that the clinical assessment and recommendations as determined by an alteration in medication type or dose made by endocrinologists for people with diabetes via videoconference are significantly different from those made through face-to-face consultation (null hypothesis). Secondary hypotheses will apply the same analytic techniques to the other aspects of diabetes consultation: (i) ordering lab tests or other diagnostic intervention; (ii) initiation of new medication(s) or dose adjustment of the previously prescribed drugs for dyslipidaemia and/or hypertension; (iii) detection or management of diabetes related complications; and (iv) referring to other specialists or arranging hospital admission.

## Methods/design

This study is a repeated-measure non-inferiority randomized controlled trial. All patients participating in the study will receive two consultations (one original consultation and one additional consultation; called paired-consultation in this paper) by two different endocrinologists. Level of agreement between the recommendations made via VC versus FTF for the same patient by two endocrinologists will be calculated. This constitutes VC-FTF paired-consultations. Since there is likely to be a certain level of clinical variability between clinicians, the level of agreement in VC-FTF paired-consultations will then be compared against the level of agreement between two endocrinologists when they consult a patient in standard clinical practice (FTF-FTF). This arrangement will determine whether any lack of agreement is likely to be a result of the videoconference modality, or just normal variation between doctors.

### Study setting and participants

This study will be conducted in the outpatient diabetes clinic of the Princess Alexandra Hospital (PAH) which is a tertiary teaching hospital in Brisbane, Australia. People with diabetes who have an appointment with an endocrinologist as a new or review case for the purpose of improving management of their diabetic condition will be approached and invited for participation. Six endocrinologists who attend the clinic routinely will visit the patients as scheduled (these are referred to as ‘routine endocrinologists’ in this paper). Another endocrinologist (referred to as the ‘research endocrinologist’ in this paper) will be employed for the purpose of this research to undertake the additional consultation for each patient who consents to participate in the trial. The additional consultation will be conducted by the research endocrinologist prior to the original consultation for each patient. However, the participant patients will receive both their consultations on the same day in the same clinic session. The routine and research endocrinologists are all specialist doctors with the same qualifications and credentials (i.e. accredited by the Royal Australian College of Physicians).

### Inclusion and exclusion criteria

Eligible participants include patients (i) with a confirmed diagnosis of diabetes, (ii) who are 18 years of age or older. Patients will be excluded if (i) they are severely ill, (ii) unable to communicate effectively (blind, deaf, mute, etc.), or (iii) speak in a language other than English if an interpreter is not available.

### Randomisation

**Figure 1:**
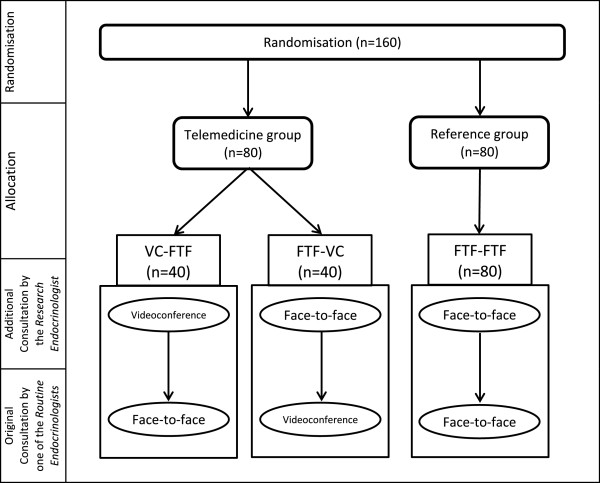
Flow diagram of randomisation and allocation to paired consultations method.

Participants will be randomly allocated in a 1:1 ratio into one of two study groups (Telemedicine or Reference group). In the Telemedicine group the participants will receive a paired-consultation in which one of the consultations is via video (FTF-VC or VC-FTF), but in the Reference group both consultations will be face-to-face (FTF-FTF). Figure 1 outlines the randomisation and allocation process. The first consultation in each paired-consultation will be provided by the research endocrinologist, whereas the second consultation will be provided by one of the routine endocrinologists of the clinic. Since the order of VC vs. FTF consultations are also randomised, there will be three potential configurations for the paired consultations of each participant (Table 1). With such design, half of the video consultations will be conducted by the research endocrinologist, and the other half by the routine endocrinologists of the clinic.

**Table 1:** Potential configurations of paired consultations for randomisation

**Group**	**Configuration**	**Consultation 1 (by the research endocrinologist)**	**Consultation 2 (by the routine endocrinologists)**
Reference (50% of participants)	1	FTF	FTF
Telemedicine (50% of participants)	2.1	VC	FTF
	2.2	FTF	VC

FTF: Face-to-Face, VC: Videoconference.

A block randomisation with the block size of eight will be used to ensure balanced representation of the participants in each group. The randomised configuration will be provided by an independent biostatistician using SAS software. Opaque sealed envelopes with sequential numbers will be used for allocating the participants into the groups.

### Informed consent and recruitment

Eligible patients will be contacted by phone prior to their appointment to explain the project and seek verbal consent. Once checked-in to the clinic for their appointment, patients who have verbally consented will be given the participant information sheet, and written consent will be obtained. Each participant will be offered two movie vouchers (valued roughly US$ 30.00) as a compensation and appreciation for participating in this study. Non-consenting or excluded patients will receive their routine service at the clinic.

### Usual care

Princess Alexandra Hospital operates three diabetes clinics a week in the outpatient clinic building. New patients are allocated a 45-minute, and review patients usually a 30-minute time slot for consultation. Wherever possible, review patients are scheduled to see the same endocrinologist they saw for their previous appointment to ensure continuity of care.

Following arrival at the clinic, the patient is received by a diabetes nurse practitioner and initial assessments such as measuring weight and blood pressure are performed. The nurse also downloads the blood glucose readings from the patient’s glucometer and updates the patient charts with the latest lab results. The patient then visits the endocrinologist. At the conclusion of the consultation, the doctor writes the progress notes and any pharmacy scripts that are required, requests pathology test(s), refers the patient to another specialist or other health professional where indicated, or arranges for hospital admission if needed. The patient is then scheduled for another follow up outpatient appointment, or care is transferred back to the referring GP with a management plan.

### Intervention

All participants, both in the Telemedicine and Reference groups, will receive an additional consultation (consultation 1) by the research endocrinologist who is employed for this study. This endocrinologist is officially qualified and credentialled to visit and manage patients at the clinic. The additional consultation will always occur prior to the original consultation (consultation 2). For the participants in the Reference group, both consultations will be face-to-face: consultation 1 by the research endocrinologist and consultation 2 by one of the routine endocrinologists of the clinic. For the participants in Telemedicine group, one consultation will be face-to-face and the other one via videoconferencing. The format of the first and second consultations (FTF-VC, or VC-FTF) for the participants in this group will be determined in the randomisation process. The final recommendation for patient management will be provided by the routine endocrinologists at the end of second consultation.

To ensure that the integrity of usual patient care is maintained for the participants in the Telemedicine group who will have their second consultation via videoconferencing (Table 1: configuration 2.2), they will be able to meet the endocrinologist face-to-face immediately after video-consultation, if required by either the endocrinologist or the patient.

### Access to patient information in video consultations

To be able to isolate the effect of videoconferencing from other factors on the outcome of each consultation, we assume that doctors would have access to the same patient information as in a face-to-face consultation. Since the endocrinologist and the patients will be in the same building for both face-to-face and video consultations, it will be possible to provide the endocrinologists with the full records of the patients in hard copy during the videoconferencing as well as access to the electronic patient record via the Queensland Health network. Similar to original consultations, the latest blood glucose readings will be downloaded from each patient’s glucometer and entered in the patient record by a practice nurse upon check-in of each patient to the clinic. That will ensure that the endocrinologists will have equivalent access to the latest blood glucose measures, in each of the paired consultations.

### Equipment and connectivity

Remote video consultation will be simulated using one video-enabled laptop dialling into the diabetes clinic telehealth studio. The telehealth studio is currently functioning and located in the same building of the clinic. For video consultations, the patient will be accompanied by a diabetes nurse educator, who will “host” the consultation and assist with aspects of clinical examination, if needed, under the direction of the endocrinologist. This arrangement closely emulates the typical situation for diabetes remote consultation by videoconference, where patients are accompanied either by a nurse or their GP.

The telehealth studio in the diabetes clinic, where the patient will sit, is equipped with a Tandberg codec 990MXP + camera unit, Sony Bravia 32” television, and an Audio-Technica microphone. This codec provides pan, tilt, zoom functions for the camera by both the local and remote parties. The endocrinologists will use a laptop with 13” screen and Cisco Telepresence Movi software ver. 4.2 (Cisco systems, San Jose, California, USA) to connect to the telehealth studio for conducting the video consultations. Both Tandberg codec and Cisco Movi are H.264 compliant and capable of high definition video (up to 1080p 30 fps) encoding and decoding. The laptop has been tested to be compatible with the Tandberg codec. This is the configuration that clinicians use when they provide remote consultations on a trip or in the facilities that dedicated VC equipment is not available. The connection will be through the existing Local Area Network (LAN) and Wireless LAN (WLAN) within the clinic building. Although the LAN bandwidth is 100 Mbps, the codec and software will be set on 384 Kbps. This bandwidth is generally regarded as the minimum connection speed for producing acceptable full screen, full motion video. Although the general connection speeds between the Queensland Health telehealth centres range from 512 kbit/s to 2.3 Mbit/s depending on site specific connections, selection of minimum required bandwidth will ensure the results of this research to be more generalizable to the countries that high speed networks are not readily available.

### Outcome measures

The outcome measure of this study is the difference in level of agreement between the two groups. Agreement will be calculated for each group (Reference group: FTF-FTF; Telemedicine group: FTF-VC or VC-FTF) and compared. For the comparison between two consultations to be accurate, it is necessary that the endocrinologists be blinded to each other’s assessment and recommendations. Part of this information can be communicated by the patient, which is inevitable, however the research endocrinologist has been requested to refrain from giving information about the assessment and treatment plan to the patient during the first consultation. Since the research endocrinologist adds nothing to the patient chart (either in hard copy or electronic records), it will ensure that the endocrinologists will be blinded to each other’s opinion on each patient.

The primary outcome measure is the level of change in patient’s medications during the consultation. Based on its impact, medication change is divided into three categories: Major, Minor, and No change (Table 2). When more than one category is applicable to a participant, the highest impact will be regarded as the overall impact of the medication change.

**Table 2:** The categories of the impact of medication change

**Medication**	**Change**	**Category**
Insulin	Initiation of insulin	Major
	Cessation of Insulin	Major
	Change in regimen (type, injection frequency)	Major
	Dose adjustment	Minor
	No change	No change
Other hypoglycaemic agents	Initiation of new drugs	Major
	Cessation of drugs	Major
	Dose adjustment	Minor
	No change	No change
Other medications (hypertension, lipids, etc.)	Initiation of new drugs	Major
	Cessation of drugs	Major
	Dose adjustment	Minor
	No change	No change

Secondary outcomes focus on performing the physical examination, detection of diabetes complications and patient satisfaction. For each video consultation, the endocrinologist will be asked about any technical problem or limitation during the video visit. This will assist in understanding the barriers of videoconferencing for consulting people with diabetes.

### Data collection

The endocrinologists will complete a questionnaire for each patient they consult [see Additional file 1]. The questionnaire will capture various elements of each consultation and comprises 16 questions in three sections: (1) Patient characteristics, (2) Procedures and findings, and (3) Recommendations. The questions have been developed based on the results of two previous studies: observing conventional face-to-face diabetes consultations, [19] and process analysis of video teleconsultation for diabetes [20]. The questionnaire has been pilot tested in four consultations and modifications are made to the questions as suggested by the endocrinologists and the researchers. The participants in the telemedicine group will also be asked to complete a patient questionnaire after their video consultation. This satisfaction questionnaire comprised 17 questions in five-point Likert-type scale asking for various aspects of videoconference session.

### Statistical methods

Demographic and baseline data will be reported as absolute numbers, percentage, and/or mean +/− SD. Percentage agreement and the weighted kappa statistic (Kw) will be used to assess inter-rater reliability between the two groups on the agreement on assessments and recommendations made by the endocrinologists [21].

### Sample size

This study will evaluate if agreement on the recommendations for the Telemedicine group is not inferior to agreement in the Reference group by more than an acceptable amount. This clinically acceptable amount of variation for diabetes consultation was set as 20% by a group of expert specialist consultants who had more than five years of telemedicine experience. The sample size is calculated based on the incidence rates of 25, 50, 25% for major change, minor change, and no change respectively in the patient medication made by the endocrinologists (significance level 5%, power 80%). It will be possible with a total of 160 participants (80 per each group) to detect any statistically significant difference for the true kappa of 0.7 and the null kappa of 0.5.

### Ethics and trial registration

Ethics approval for this study has been obtained from the Human Research Ethics Committee of Queensland Health (HREC/11/QPAH/645 – 12/03/2012) as well as The University of Queensland School of Medicine (2011-SOMILRE-0022 – 4/05/2012). This study is also registered by Australian New Zealand Clinical Trials Registry (ANZCTR) as a randomized controlled trial (ACTRN12612000315819, 20/03/2012).

## Discussion

Results of the proposed study will provide an important and novel insight into provision of clinical consultation remotely to patients with diabetes by endocrinologists. It will investigate whether videoconferencing is as reliable and safe as face-to-face encounter for management of diabetes. To our best knowledge, this is the first RCT looking at safety of videoconferencing for specialty consultation of diabetes.

Global prevalence of diabetes has been estimated to increase from 8.3% in 2011 to 9.9% in 2030 among the adult population [1]. During this period, developing countries will have a 69% increase in prevalence of diabetes whereas this increase will be 20% for developed countries [22]. Although this study has been designed and will be conducted in Australia which is categorized as a high-income country, the proposed intervention has potential to be adopted in all countries that meet the minimum technical requirements for a videoconferencing with quality accepted for clinical purposes. The World Health Organization (WHO) has recommended the incorporation of newer technologies, such as telecommunications, into the health care system to improve access to health services in resource limited countries [23]. Many health centres in low-middle income countries already have Internet connection that is a prerequisite in most of telemedicine interventions including video teleconsultation, which has been proposed here.

Consultants’ style of clinical practice might be different from their regular practice while they know they are involved in a research study (Hawthorne effect). However, this potential effect will equally affect both Reference and Telemedicine groups. Consultants are also required to fill in a questionnaire for each consultation in this study. Items included in the questionnaire that are derived from observing routine consultations in the outpatient clinic of a teaching hospital, can act as a checklist and serve as a decision support system that possibly improve the process of the consultation, but again this effect will be equally distributed among the two groups.

Many hospitals and clinics are currently utilizing electronic patient records, either as a substitution to the traditional paper-based patient’s records or as a complement to them. In this study the endocrinologists will have access to the patient’s complete medical records equally in both face-to-face and video consultations. This is not the case in the real world, except for the settings in which the patient records are fully electronic and accessible via network, and if there is some additional information on hard copy, that information would be sent to the tele-consultant before or during the consultation.

A limitation of the study is the inability to randomise the order of the endocrinologists that the patient will see and which endocrinologist would provide the final treatment recommendation. This is not able to be performed due to practical issues involved around ensuring the smooth running of a busy outpatient clinic as there is not time for both endocrinologists to discuss each patient and provide a collaborative management plan.

Despite the rapid growth in telemedicine services in Australia and official adoptation of videoconferencing as a mode of delivery for clinical consultations in Australian health system and worldwide, little has been published on safety and reliability of videoconferencing for remote consultation of people with diabetes. This study will fill in the gap of research in the field of telemedicine for diabetes, and may serve to guide the application of telemedicine to the management of other chronic diseases.

## Abbreviations

ICT: Information and communications technology; VC: Videoconference, videoconferencing; FTF: Face-to-face.

## Competing interests

The authors declare that they have no competing interests.

## Authors’ contributions

FF contributed to the study design and drafted the manuscript. MMK and LG conceived the original study design. FF, LG and AR participated in conceptualisation of the intervention and developing the outcome measures. MMK participated in the development of the protocol and revised the manuscript. All authors reviewed and approved the final manuscript.

## Pre-publication history

The pre-publication history for this paper can be accessed here:

http://www.biomedcentral.com/1472-6947/14/11/prepub

## Supplementary Material

Additional file 1 Analysis of endocrinology consultation. Click here for file

## References

[B1] Whiting DR Guariguata L Weil C Shaw J IDF diabetes atlas: global estimates of the prevalence of diabetes for 2011 and 2030 Diabetes Res Clin Pract 2011 94 3 311 321 10.1016/j.diabres.2011.10.029 22079683

[B2] The Diabetes Control and Complications Trial Research Group The effect of intensive treatment of diabetes on the development and progression of long-term complications in insulin-dependent diabetes mellitus N Engl J Med 1993 329 14 977 986 8366922 10.1056/NEJM199309303291401

[B3] Bouma M Dekker JH van Eijk JT Schellevis FG Kriegsman DM Heine RJ Metabolic control and morbidity of type 2 diabetic patients in a general practice network Fam Pract 1999 16 4 402 406 10.1093/fampra/16.4.402 10493712

[B4] Dunn NR Bough P Standards of care of diabetic patients in a typical English community Br J Gen Pract 1996 46 408 401 405 8776910 PMC1239691

[B5] Ward MM Access to care and the incidence of end-stage renal disease due to diabetes Diabetes Care 2009 32 6 1032 1036 10.2337/dc09-0017 19460914 PMC2681017

[B6] Zhang X Bullard KM Gregg EW Beckles GL Williams DE Barker LE Albright AL Imperatore G Access to health care and control of ABCs of diabetes Diabetes Care 2012 35 7 1566 1571 10.2337/dc12-0081 22522664 PMC3379598

[B7] Charles BL Telemedicine can lower costs and improve access Healthc Financ Manage 2000 54 4 66 69 10915354

[B8] Field MJ Grigsby J Telemedicine and remote patient monitoring JAMA 2002 288 4 423 425 10.1001/jama.288.4.423 12132953

[B9] Kayser K Interdisciplinary telecommunication and expert teleconsultation in diagnostic pathology: present status and future prospects J Telemed Telecare 2002 8 6 325 330 10.1258/135763302320939202 12537919

[B10] Barneveld Binkhuysen FH Ranschaert ER Teleradiology: evolution and concepts Eur J Radiol 2011 78 2 205 209 10.1016/j.ejrad.2010.08.027 20869183

[B11] High WA Houston MS Calobrisi SD Drage LA McEvoy MT Assessment of the accuracy of low-cost store-and-forward teledermatology consultation J Am Acad Dermatol 2000 42 5 Pt 1 776 783 10775853 10.1067/mjd.2000.104519

[B12] Joseph B Hadeed G Sadoun M Rhee PM Weinstein RS Video consultation for trauma and emergency surgical patients Crit Care Nurs Q 2012 35 4 341 345 10.1097/CNQ.0b013e318266c2f2 22948367

[B13] Barton C Morris R Rothlind J Yaffe K Video-telemedicine in a memory disorders clinic: evaluation and management of rural elders with cognitive impairment Telemed J E Health 2011 17 10 789 793 10.1089/tmj.2011.0083 22023458

[B14] Hakansson S Gavelin C What do we really know about the cost-effectiveness of telemedicine? J Telemed Telecare 2000 6 Suppl 1 S133 136 10.1258/1357633001934438 10793998

[B15] Verhoeven F Tanja-Dijkstra K Nijland N Eysenbach G van Gemert-Pijnen L Asynchronous and synchronous teleconsultation for diabetes care: a systematic literature review J Diabetes Sci Technol 2010 4 3 666 684 20513335 10.1177/193229681000400323 PMC2901046

[B16] Siriwardena LS Wickramasinghe WA Perera KL Marasinghe RB Katulanda P Hewapathirana R A review of telemedicine interventions in diabetes care J Telemed Telecare 2012 18 3 164 168 10.1258/jtt.2012.SFT110 22362832

[B17] Martin-Khan M Flicker L Wootton R Loh PK Edwards H Varghese P Byrne GJ Klein K Gray LC The diagnostic accuracy of telegeriatrics for the diagnosis of dementia via video conferencing J Am Med Dir Assoc 2012 13 5 487 e419 e419-424 22572552 10.1016/j.jamda.2012.03.004

[B18] Piaggio G Elbourne DR Pocock SJ Evans SJ Altman DG Group C Reporting of noninferiority and equivalence randomized trials: extension of the CONSORT 2010 statement JAMA 2012 308 24 2594 2604 10.1001/jama.2012.87802 23268518

[B19] Fatehi F Gray LC Russell AW A Clinimetric Study of Outpatient Diabetes Consultations: The Potential for Telemedicine Substitution Diabetes Technol Ther 2013 16 1 8 14 24156361 10.1089/dia.2013.0213

[B20] Fatehi F Gray LC Russell AW Telemedicine for clinical management of diabetes -- a process analysis of video consultations J Telemed Telecare 2013 19 7 379 382 10.1177/1357633X13506524 24218349

[B21] Landis JR Koch GG The measurement of observer agreement for categorical data Biometrics 1977 33 1 159 174 10.2307/2529310 843571

[B22] Shaw JE Sicree RA Zimmet PZ Global estimates of the prevalence of diabetes for 2010 and 2030 Diabetes Res Clin Pract 2010 87 1 4 14 10.1016/j.diabres.2009.10.007 19896746

[B23] UNAIDS Resource needs for an expanded response to AIDS in low-and middle-income countries 2005 Geneva: WHO

