# Supplementary material for: Design of a randomized, non-inferiority trial to evaluate the reliability of videoconferencing for remote consultation of diabetes
Source: BMC Med Inform Decis Mak. 2014 Feb 14;14:11. doi: 10.1186/1472-6947-14-11 (PMC3925960; doi:10.1186/1472-6947-14-11)
Supplement: Additional file 1 — Analysis of endocrinology consultation. [file 1472-6947-14-11-S1.pdf]

Please return to:
Farhad Fatehi
Centre for Online Health
Tel: 0430 992 014
Fax : (07) 3176 6945
Email: f.fatehi@uq.edu.au

Analysis of endocrinology consultation

Clinician Name: .... Patient ID: .... Consultation Date: .... Mode: ❍in-person / ❍VC

Times: Case preparation ……. minutes; Consultation: from .... to ....; writing report: ………… minutes

I. Patient characteristics:

1. Year of birth: ....
2. Gender: 0❍Female 1❍Male
3. How far from the clinic is the patient living?
   1❍<20 KM 2❍20-100 KM 3❍>100 KM
4. Prior to this consultation, which of the following treatments were prescribed?
   a. ❑ Diet
   b. ❑ Exercise
   c. ❑ Oral diabetic medication
   d. ❑ Byetta
   e. ❑ Insulin

II. Procedures and findings:

5. Latest HbA1c: ………….
6. How was the physical examination performed?
   0❍ Not performed *(Go to Question 9)*
   1❍ By the endocrinologist
   2❍ By the endocrinologist, with nursing assistance
   3❍ Remotely by the VC host (e.g. nurse)
7. Which examination(s) was performed?
   a. ❑Feet – general inspection
   b. ❑Feet – assessment of pulses
   c. ❑Feet – neuropathy exam
   d. ❑Eyes
   e. ❑Injection sites
   f. ❑Cardiovascular
   g. ❑Respiratory
   h. ❑Abdominal
   i. ❑Neurological
   j. ❑Other ....
8. Was any new important sign detected in patient's physical examination which in your opinion would influence management?
   a. ❑Feet – general inspection
   b. ❑Feet – assessment of pulses
   c. ❑Feet – neuropathy exam
   d. ❑Eyes
   e. ❑Injection sites
   f. ❑Cardiovascular
   g. ❑Respiratory
   h. ❑Abdominal
   i. ❑Neurological
   j. ❑Other *(Please specify)* ....
   ....
   ....
   ....
9. Does the patient have any of these complications?
   a. ❑Retinopathy
   b. ❑Nephropathy
   c. ❑Neuropathy
   d. ❑Diabetic foot ulcer
   e. ❑Ischemic Heart Disease
   f. ❑Other ....
10. What is your assessment of the patient's condition?
   1❍ The patient's condition is satisfactory
   2❍ More investigations are needed
   3❍ There is a need for change in management plan

IV. Recommendations:

11. Did you advise any life style change?
   a. ❑Healthy diet
   b. ❑Physical activity
   c. ❑Sport
   d. ❑Other ....
12. Did you order any of the following investigations?
   a. ❑ Lab test(s) ....
   b. ❑ Imaging(s) ....
   c. ❑ Other ....
   d. ❑ No investigation was ordered
13. Did you change the patient medication?
   a. ❑ No; current treatment plan confirmed
   b. ❑ Yes, <u>Insulin</u>
      i. ❍ Initiation
      ii. ❍ Dose adjustment
      iii. ❍ Change in regimen (type, frequency)
      iv. ❍ Cessation
   c. ❑ Yes, <u>other hypoglycaemic</u> agents
      i. ❍ Initiation
      ii. ❍ Dose adjustment
      iii. ❍ Change in regimen (type, frequency)
      iv. ❍ Cessation
   d. ❑ Yes, <u>Anti-hypertensive</u> drugs
      i. ❍ Initiation
      ii. ❍ Dose adjustment
      iii. ❍ Change in regimen (type, frequency)
      iv. ❍ Cessation
   e. ❑ Yes, <u>Lipid</u> drugs
      i. ❍ Initiation
      ii. ❍ Dose adjustment
      iii. ❍ Change in regimen (type, frequency)
      iv. ❍ Cessation
   f. ❑ Yes, Other: ………………………………………
      i. ❍ Initiation
      ii. ❍ Dose adjustment
      iii. ❍ Change in regimen (type, frequency)
      iv. ❍ Cessation

14. Did you refer the patient?
   a. ❑ No.
   b. ❑ Yes; the patient was referred
      i. ❍ back to the GP
      ii. ❍ to another specialist
      iii. ❍ to an allied health professional
      iv. ❍ for hospital admission
      v. ❍ other ....

15. Did you arrange any follow-up?
   a. ❍ Yes, at this clinic, in ………….. weeks
   b. ❍ Yes, at another clinic
   c. ❍ No; Follow-up was not needed
   d. ❍ No; Follow-up was not possible
   e.

16. Please indicate any problem you might have with videoconferencing (if the consultation was via video).

………
………
………
………
………
………
………
………

Thank you!

❍Denotes mutually exclusive options - ❑Denotes potentially inclusive options

*High Definition Videoconferencing is provided by means of special equipment with capability of zooming, panning, tilting of camera and high quality video and audio
